# A systematic review of psychological interventions in adults with pulmonary hypertension: Is the evidence‐base disproportionate to the problem?

**DOI:** 10.1111/crj.13685

**Published:** 2023-08-15

**Authors:** Gregg H. Rawlings, Barbora Novakova, Iain Armstrong, Andrew R. Thompson

**Affiliations:** ^1^ School of Social Sciences Nottingham Trent University Nottingham UK; ^2^ Health and Wellbeing Service Sheffield IAPT, Sheffield Health and Social Care NHS Foundation Trust Sheffield UK; ^3^ Sheffield Pulmonary Vascular Disease Unit Royal Hallamshire Hospital, Sheffield Teaching Hospitals NHS Foundation Trust Sheffield UK; ^4^ South Wales Clinical Psychology Training Programme Cardiff and Vale University Health Board and Cardiff University Cardiff UK

**Keywords:** anxiety, cognitive behavioural therapy, depression, quality of life, treatment

## Abstract

**Introduction:**

Our understanding of the psychological impact of living with pulmonary hypertension (PH) is growing, particularly for how anxiety and depression present in this group. There is evidence for the use of psychological interventions in other chronic lung conditions; however, trials focusing on adults with PH have yet to be subject to a systematic review.

**Methods:**

We systematically searched four databases for evidence examining the effectiveness of psychological interventions for adults with PH. A narrative approach has been used to present findings.

**Results:**

Overall, 186 unique articles were identified of which four were suitable. Data was gathered from 143 individuals. Cognitive behavioural therapy (CBT), metacognitive therapy (MCT) or counselling were investigated. All three studies reported a significant reduction in anxiety and depression, and some secondary outcomes were also associated with change.

**Discussion:**

The evidence supporting the use of psychological therapies for adults with PH is promising, although limited. There is an urgent need for more research in this area to better understand how we can support this clinical group.

## Graphical abstract

Our understanding of the psychological impact of living with pulmonary hypertension (PH) is growing. Evidence exists for the use of psychological interventions in chronic lung conditions; however, trials involving adults with PH have not been subject to a systematic review. We show that although the evidence is limited, the findings are promising and there is a need for more research.

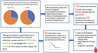

## 1 BRIEF REPORT

Pulmonary hypertension (PH) is a group of serious and life‐limiting conditions.^1^ Research into PH has primarily focused on reducing early mortality and morbidity rates, and it is only recently that the psychological, emotional and social impact of PH has been more extensively examined. Symptoms associated with anxiety and depression, which have also been shown to be correlated with health‐related quality of life in people with PH,^2^, ^3^ appear to have received the greatest attention. Rates of mood disorders have been described as alarming, with observed figures being considerably high (rate of depression: 95% CI: 20.5%–36.8%; anxiety: 95% CI: 28.7%–46.4%).^4^

Our understanding of how anxiety, depression and other psychological difficulties interact with PH‐related symptoms is currently developing. The available studies indicate that disease specific factors alone are unable to fully account for the reported impact^5^, ^6^, ^7^ with physiological measures of functioning tending to be non‐significant risk factors for depression or anxiety.^4^ There is also a need for studies to identify the underlying psychological mechanisms implicated in adjustment to living with the condition. This would enable the development of targeted psychosocial interventions that are likely to be effective in reducing the impact of living with PH.

There is some evidence for the use of psychological therapies for anxiety and depression in people with other chronic lung conditions.^8^ Unfortunately, the availability of such interventions in people with PH appears to be limited.^9^ Further, whilst several studies exist, they have not been subject to a systematic review, with previous attempts to review psychological interventions failing to utilise appropriate review methods^10^, ^11^ or including non‐psychological interventions.^4^ We report on our systematic review of the evidence examining the effectiveness of psychological interventions for adults with PH.

We performed a systematic search in February 2023 of four databases: PsycINFO, MEDLINE, Cochrane library and CINAHL. Search terms were informed by relevant articles known to the authors^12^, ^13^ (Table 1). Inclusion and exclusion criteria are reported in Table 2. Studies were included if they examined any patient‐report outcome measure in response to a psychological intervention. Participants must have been adults (>18 years) and diagnosed with any form of PH.

**TABLE 1:** Search terms.

Term 1	AND	Term 2
Pulmonary hypertension OR pulmonary arterial hypertension		Psychotherapy OR psychological therapy OR psychological treatment OR psychological intervention OR counselling OR cognitive behaviour therapy*

**TABLE 2:** Inclusion and exclusion criteria.

	Inclusion	Exclusion
Participants	Adults (18 years or older) living with any form of PH	Studies where the mean age of the sample is <18 years, and studies involving people who are not living with PH.
Intervention	Receiving a psychological intervention—this may be a stand‐alone intervention or combined with treatment as usual.	Receiving an intervention that is not psychological therapy, for example, progressive muscle relaxation, breathing exercises, yoga.
Comparison	Any comparison group, that is, treatment as usual, wait list group.	‐
Outcome	Self‐report measure of psychological distress.	Studies that only measure a psychological construct, that is, maladaptive cognitions and behaviours, or a non‐psychological distress, that is, breathing difficulties, healthcare utilisation or measures of physical ability.
Format	Any format, that is, grey literature, book chapter. Written in English. Published in a peer reviewed journal. No date limits were used.	Articles that do not present new data, such as reviews. Articles not published in English.

Abbreviation: PH, pulmonary hypertension.

**FIGURE 1:**
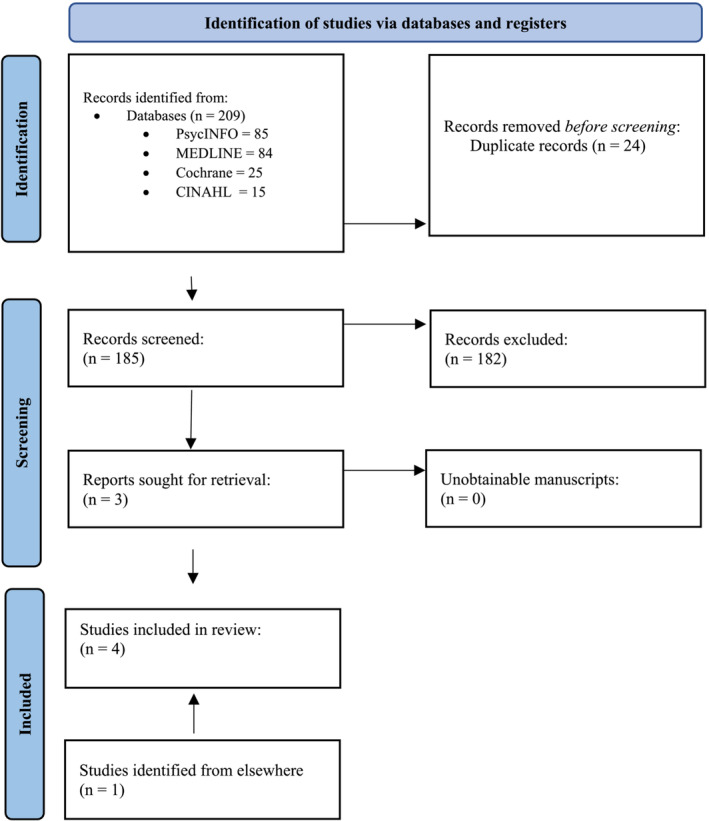
Prisma flow diagram.

Title and abstract of articles identified were first screened. Most studies were excluded at this stage as they did not investigate PH or were not evaluating a psychological intervention. If relevant, articles were then subject to a full‐text review. Forward and backward searchers of included articles were performed, including searches for articles in previous reviews,^4^, ^10^ which generated one additional study (Figure 1).

Relevant data based on participant, intervention, comparison group and outcome (PICO) were extracted from each article (see Table 3). Given the small number of studies and heterogeneity in research methods, a narrative approach was deemed as most suitable to present the findings.

**TABLE 3:** Summary of studies examining patient‐reported outcomes in adults with PH in response to psychological interventions.

Author	Country	Study design	Sample size	Participants	Intervention	Comparison group	Self‐reported measurements	Outcome
Bussotti (2016)	Italy	Observational study	*N* = 15	*N* = 15 Mean age 45 years, Female 87%, 100% Group 1 PAH, 60% class II, 40% class III, Mean time since diagnosis of PH 23 months. Outpatient setting	CBT—4‐10 sessions per patient over 4 weeks focused on anxiety, depression, and panic attacks. Interventions also included aerobic training, resistance exercises inspiratory muscle training and slow breathing sessions.	‐	Pre‐ and post‐treatment. Anxiety and depression (HADS), and quality of life (EQ‐5D and EQ‐VAS).	Wilcoxon's signed‐rank test revealed a significant reduction in anxiety (*p* < 0.01) and depression (*p* < 0.05) with large effect sizes (0.81–0.63, respectively). Significant improvement in EQ‐5D and EQ‐VAS (*p* < 0.01) with large effect sizes (0.79–0.85, respectively).
Rawlings (2022)	UK (sample recruited from UK, Canada, India, USA, the Netherlands and Australia	Pilot RCT	*N* = 77 (37 received psychological therapy)	*N* = 37 Mean age 48.2 years, female 95%, White 68%, Employed 38%. All forms of PH; most common idiopathic PH 51%, 32% class III Mean years since diagnosis of PH 9.5 Recruited from the community	CBT—4‐week self‐help intervention focused on anxiety in PH	Wait list	Pre‐ and post‐ treatment, and one‐month follow‐up. Anxiety (GAD‐7), depression (PHQ‐9), HRQoL (emPHasis‐10), breathing difficulties (D‐12), self‐mastery (self‐mastery scale), cognitive and behaviours related to anxiety and depression (CBP‐Q)	2 × 3 mixed ANOVA revealed significant differences in anxiety (*p* = 0.017, Cohen's f = 0.28), depression (*p* = 0.048, Cohen's f = 0.24)) and CBP‐Q (*p* = 0.001, Cohen's f = 0.39) between the two groups in favour of CBT. No significant differences observed in other measures.
Tarantino (2020)	Italy	Clinical trial	*N* = 50 (24 received psychological counselling)	All had PAH. Data not provided for people in the intervention group. Recruited from hospital setting	Psychological counselling—four sessions every 2 weeks focusing on anxiety and depression. Delivered by psychologist with expertise in systemic psychotherapy during routine visits to the centre.	Received one session or no sessions of psychological counselling	Pre‐ and post‐ treatment, quality of life, anxiety/depression, and self‐perceived health state (EQ‐5D and EQ‐VAS)	One way ANOVA revealed a significant effect on anxiety/depression scale (*p* < 0.001) showing a reducing in those in therapy. Z‐test between groups revealed a significant difference for self‐perceived health state and anxiety/depression scale post treatment (*p* < 0.001)
Winter (2020)	Germany	Case report	*N* = 1	34‐year‐old female, hereditary PAH reported to be newly diagnosed. Inpatient setting.	MCT focusing on adjustment disorder. Four weekly sessions of MCT lasting 50 min each.	‐	Pre‐ and post‐treatment and 6 weeks follow up. Anxiety and depression (HADS), metacognitive questionnaire (MCQ‐30)	Pre versus post, reduction of 54% in anxiety, 57% in depression and 55% in MCQ‐30. Pre versus follow up 100% reduction of anxiety, 86% in depression, 55% in MCQ‐30.

Abbreviations: CBP‐Q, cognitive behavioural processes questionnaire; CBT, cognitive behavioural therapy; D12, dyspnoea 12; GAD‐7, generalised anxiety disorder 7; HADS, hospital anxiety and depression scale; HRQoL, health related quality of life; MCQ‐30, metacognitions questionnaire; MCT, metacognitive therapy; N, number of participants; PAH, pulmonary arterial hypertension; PH, pulmonary hypertension; PHQ‐9, patient health questionnaire‐9; RCT, randomised control trial; UK, United Kingdom; USA, United States of America.

Four studies were identified as suitable (Table 3). All were published after the year 2016. Data was collected from 143 individuals with PH—three studies focused on pulmonary arterial hypertension (PAH), whereas the other included all forms of PH. Participants were judged to be representative of the target population; three samples were recruited from hospital settings in Europe, and the other was an international study recruiting adults from the community.

Cognitive behavioural therapy (CBT),^12^, ^14^ metacognitive therapy (MCT)^15^ and unspecified counselling^13^ were evaluated using a pre‐ and post‐intervention design; two studies provided a follow‐up (4 and 6 weeks). Interventions focused on self‐reported outcomes including, anxiety (CBT), anxiety and depression (unspecified counselling), and adjustment disorder (MCT). However, one intervention reported their primary endpoint was change in peak oxygen consumption (peak VO_2_); suggesting changes in mental health were a secondary aim.^14^ Three studies used interventions delivered by a healthcare professional, the other was a self‐help programme. Only one study reported how treatment adherence was assessed in patients; no study reported on therapists' adherence to treatment modality.

Two studies included a control group; the other studies were an observational trial or a case study. All studies measured anxiety and depression; other health‐related measures and psychological processes were also assessed. Although for the most part measures were appropriate given the research aims, it is important to recognise that they were typically screening tools and did not aim to diagnose individuals.

A significant reduction in anxiety and depression was observed in all samples. There was some evidence to suggest anxiety and depression did not improve in people with PH who did not receive a psychological intervention.^12^, ^13^ Change in self‐perceived health status including quality of life was also observed.^13^, ^14^ Improvement in psychological mechanisms that are considered to underlie difficulties with mental health was observed in two studies—cognitive and behavioural process^5^ and metacognitions.^15^ This appears to be consistent with findings reported elsewhere,^2^, ^5^, ^7^ which have demonstrated psychological processes that are related to subjective measures of wellbeing and therefore a possible target for intervention. This has included coping styles, cognitions, behaviours and self‐compassion. Any changes in patient reported outcomes were also maintained at follow‐up. Although not all self‐reported outcome measures were associated with significant change, including dyspnoea, which is a cardinal symptom of PH, they should only be viewed as secondary outcomes as interventions did not target them specifically.

Individuals who scored a greater severity of symptoms were more likely to engage in therapy^13^ and less likely to drop out.^12^ It may be that this group have more of a need for psychological intervention and motivated to engage. Two studies reported on attrition demonstrating a relatively low rate of 18.9%^12^ and 6%^14^ in those receiving CBT. Indeed, there is limited evidence on the acceptability of mental health support in people with PH; a qualitative study interviewing 24 individuals with PAH found some were not open to receiving support. However, counselling or therapy was wanted by those reporting difficulties with coping with their worries and emotions, as well as individuals who received limited support from others or experiencing additional life challenges.^16^

The importance of integrating treatments for physical and mental health has long been recognised.^17^ A large body of evidence exists highlighting the reciprocal relationship between physical and mental health, acknowledging that living with a long‐term health condition is associated with higher prevalence of mental health problems. Similarly, those with psychological problems, including anxiety and depression, are more likely to experience difficulties managing their health conditions; however, this has yet to be examined in people with PH. Provision of targeted psychological support integrated within health care services is therefore essential for improving outcomes.

Advancements in how PH is diagnosed and treated have resulted in people living longer with the condition. There is now a need to extend treatment beyond the physical symptoms, as research has shown this group are significantly impacted by the condition.^3^, ^18^ Although the increased risk of mood disorders in this clinical group is well recognised, the evidence for treating such psychological distress using therapy is limited. It is clear that more research is needed to help address the gap between the problem and evidence‐base. Although data gathered from research trials are important, evidence collected from routine clinical practice are essential in helping to build the evidence. In addition to using patient reported outcomes, research should focus on patient's experiences of engaging in psychological treatments. This information can be used to glean insight into the acceptability and feasibility of interventions and improve many aspects of treatment.

This is the first systematic review examining psychological interventions for emotional difficulties in people with PH. It is a limitation that a narrative approach was used to synthesise the results; however, at this time, due to the limited data and heterogeneity in methods, samples, interventions and measures, a meta‐analysis would not be appropriate. Articles were screened and data were extracted by one researcher whereas steps could have been taken to protect against bias and human error. Despite this, the evidence is promising for the use of such therapies, but importantly, also highlights the urgent need for more research and better patient involvement and access.

## AUTHOR CONTRIBUTIONS

**Gregg H. Rawlings** developed the concept of the review. He developed the protocol, performed the systematic review, data extraction and analysis and write up. He approved the final manuscript for submission. **Barbora Novakova** was involved in the concept of the review. She provided feedback on an earlier version of the review and approved the final manuscript for submission. **Iain Armstrong** was involved in the concept of the review. He provided feedback on an earlier version of the review and approved the final manuscript for submission. **Andrew R. Thompson** was involved in the concept of the review. He provided feedback on an earlier version of the review and approved the final manuscript for submission.

## ACKNOWLEDGEMENTS

This research has received no external funding.

## CONFLICT OF INTEREST STATEMENT

Dr Rawlings has received payment from Janssen‐Cilag Ltd for a presentation on depression and pulmonary hypertension (PH).

## ETHICS STATEMENT

Ethical approval was not required for this article and therefore was not obtained.

## Data Availability

Data sharing is not applicable to this article as no new data were created or analyzed in this study.
